# Anterior Segment Changes in Patients With Glaucoma Following Cataract Surgery

**DOI:** 10.7759/cureus.58703

**Published:** 2024-04-21

**Authors:** Anna I Kouroupaki, George I Triantafyllopoulos, Evangelos Pateras, Costas H Karabatsas, Athina Plakitsi

**Affiliations:** 1 Biomedical Sciences Department, Course of Optics and Optometry, University of West Attica, Athens, GRC; 2 Ophthalmology Department, 'Korgialenio-Benakio' Hellenic Red Cross General Hospital, Athens, GRC

**Keywords:** ophthalmic viscosurgical device (ovd), trabecular-iris space area (tisa), angle-opening distance (aod), anterior chamber angle (aca), central corneal thickness (cct), axial length (al), anterior segment optical coherence tomography (as-oct), optical coherence tomography (oct), primary open-angle glaucoma (poag)

## Abstract

This prospective observational study investigates the impact of cataract surgery on anterior segment parameters in nonglaucomatous and primary open-angle glaucoma (POAG) eyes, utilizing anterior segment optical coherence tomography (AS-OCT). The study involved 42 Caucasian patients, divided into a control group and a POAG group. Comprehensive ophthalmic examinations were performed along with AS-OCT imaging and biometry preoperatively, as well as on one day, one week, and one month following cataract surgery. The results showed significant post-operative changes in anterior chamber depth (ACD) and angle width in both groups, suggesting that cataract surgery may influence the structural parameters associated with glaucoma risk and management. Specifically, a marked increase in ACD and improvement in angle-opening distances were observed, highlighting the potential of cataract extraction to alter intraocular fluid dynamics favorably. Despite these changes, the study noted an initial spike in increased intraocular pressure (IOP) in POAG patients immediately post-operative, which stabilized during follow-up. For the control group, IOP showed gradually reducing IOP values in the follow-up visits. The findings underscore the role of advanced imaging technologies in understanding glaucoma's pathophysiology and the potential benefits of cataract surgery in glaucoma patients. The study advocates for further research with a larger, more diverse patient population and extended follow-up to explore the long-term implications of cataract surgery on glaucoma dynamics, emphasizing the importance of personalized management and treatment strategies particularly for glaucoma patients.

## Introduction

Glaucoma is one of the leading causes of blindness worldwide, especially among older adults. It is estimated to affect 60 million people globally, with the number expected to rise due to the aging population [1]. It is a disease, characterized by damage to the optic nerve, often associated with increased intraocular pressure (IOP), leading to progressive and permanent vision loss if not adequately treated.

The pathogenesis of glaucoma is multifactorial, involving both mechanical and vascular factors that contribute to optic nerve damage. The most prevalent form of the condition is primary open-angle glaucoma (POAG), which is particularly insidious as it typically progresses without symptoms until significant vision loss has occurred [2]. There is evidence that anterior segment parameters have a significant role in the pathophysiology of several types of glaucoma, including POAG [3-6]. It is important to note that, following an uneventful phacoemulsification surgery, anterior segment variables tend to change, including anterior chamber depth and angle width. Whether such changes may be beneficial for patients with glaucoma or for patients at risk for developing glaucoma is not quite clear.

Modern imaging technology, such as optical coherence tomography (OCT), has improved the ability to detect and analyze glaucomatous damage [7,8]. POAG manifestations involving the primary optic nerve and the retinal nerve fiber layer and examination of the associated parameters have become a standard of care for monitoring patients with glaucoma [9]. However, modalities such as the anterior segment OCT (AS-OCT) enable the assessment and visualization of the structures of the anterior segment of the eye [10]. It is particularly useful for detailed examination and analysis of the iridocorneal angle, the corneal thickness, and the intraocular lens (IOL) position. The AS-OCT can also be used to examine the iris configuration, which might have implications for disease management [11,12].

While previous research has acknowledged the impact of cataract surgery on anterior chamber anatomy and physiology, there remains a critical knowledge gap, particularly concerning its effects on patients with glaucoma. Understanding these changes is imperative for optimizing postoperative outcomes, including IOP reduction [13]. Additionally, investigating these effects in patients with open angles, where mechanisms for IOP reduction are poorly understood, is essential for developing targeted management strategies and improving treatment efficacy in this specific patient cohort. Based on the above, we designed a study that explores anterior chamber parameters' in both glaucomatous and non-glaucomatous eyes following cataract surgery. This study aims to utilize the AS-OCT to investigate how cataract surgery influences anterior segment structures in glaucoma patients, thereby contributing to a deeper understanding of glaucoma management post-operatively.

## Materials and methods

This prospective observational study was conducted in the Ophthalmology Department of Korgialenio - Benakio General Hospital in Athens, Greece. The study protocol was in accordance with the Declaration of Helsinki and was approved by the Research Ethics Committee of the University of West Attica (approval protocol number: 36659; date: April 27, 2021). The purpose of this study was explained to all participants and signed written consent was obtained. Forty-two patients were included.

In this study, patients with POAG, defined as an eye with an open angle, elevated IOP, and glaucomatous optic neuropathy such as optic nerve head excavation or thinning of the neuroretinal rim and corresponding visual field defects were considered for further analysis.

Eyes with a history of angle-closure attack and/or previous laser iridotomy were excluded for the purpose of this study. Moreover, in cases where angle obstruction by the peripheral iris was observed in gonioscopy, such as peripheral anterior synechiae, iris whirling, or excessive pigment deposition on the trabecular surface, patients were also excluded. Eyes with a history of intraocular inflammation or uveitis, previous intraocular surgery, or other intraocular pathology were excluded.

Patients underwent complete ophthalmic examination, including best-corrected visual acuity (BCVA), IOP measurement, gonioscopy, dilated fundus examination, AS-OCT, axial length (AL), and central corneal thickness (CCT) measurements. The number of ocular hypotensive medications used by each patient was reported prior to surgery. All operations were performed under topical anesthesia by the same surgeon (AK). In all eyes, a 2.75 mm clear corneal incision through a temporal approach was created. Following the incision, a continuous curvilinear capsulorhexis with an approximate 5.5 mm diameter was performed. The hydrodissection was followed by phacoemulsification of the nucleus and cortex aspiration. The lens capsule was inflated with an ophthalmic viscosurgical device (OVD), and the foldable IOL was placed in the capsular bag. The corneal wound was not sutured. There were no intraoperative or post-operative complications to be reported for any patient.

AL and CCT measurements were taken with the IOL Master 700 (Carl Zeiss Meditec AG, Jena, Germany), while AS-OCT images were obtained using the RT Vue XR Avanti device (Optovue Inc., Fremont, CA). Raw images were taken by the spectral domain OCT with the add-on lens of the corneal adaptor module and were further analyzed by two experienced graders. For image capture, the pupil was undilated, and the patient was asked to sit and fixate on an indicator in the AS-OCT. Images of the nasal and temporal angle quadrants (0° and 180° meridians) were captured until the centration and quality were enough to analyze. For the purpose of this work, the average number between nasal and temporal measurements was used for further analysis. Measures were taken one day, one week, and one month after the operation.

**Figure 1:**
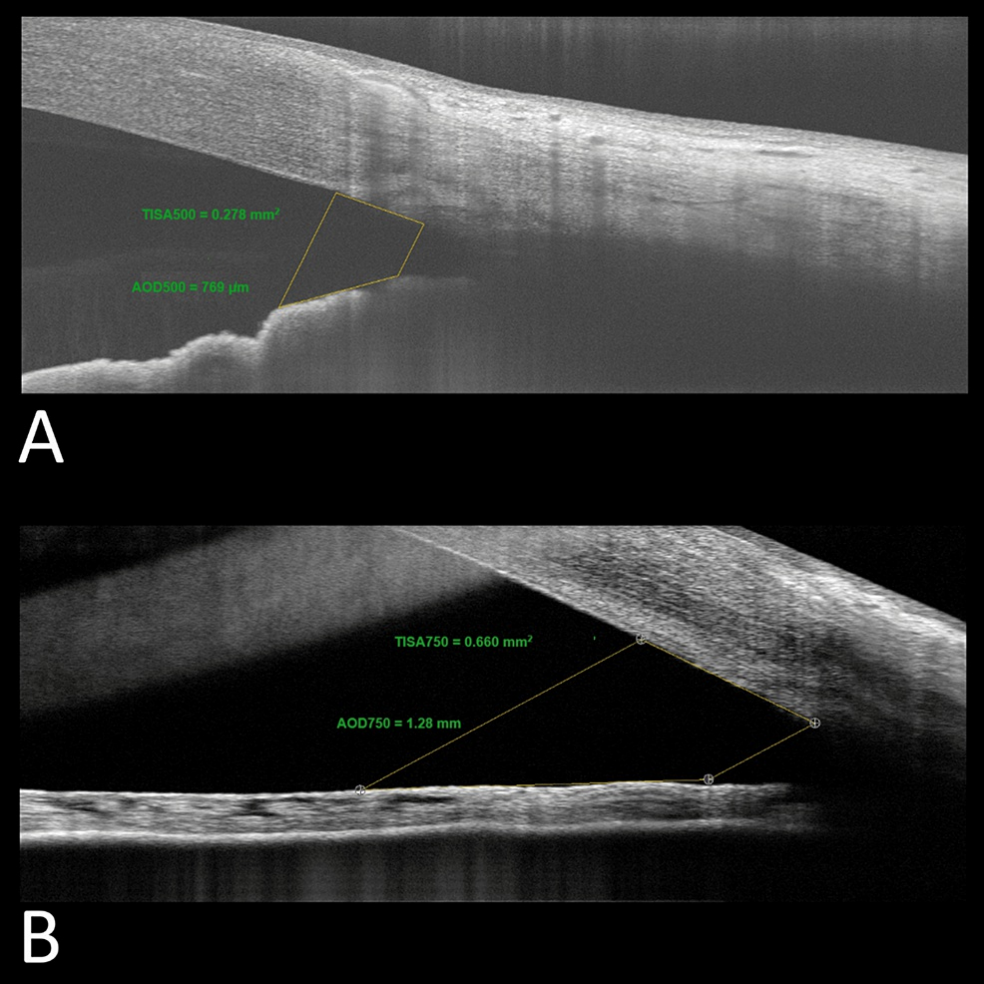
(A) TISA500 and AOD500 measured pre-operatively. (B) TISA750 and AOD750 measured post-operatively. Note the flat iris configuration that applies to angle widening after cataract removal. TISA, trabecular-iris space area; AOD, angle-opening distance

Anterior chamber and angle parameters were measured: the anterior chamber angle (ACA), defined as the angle between the iris tangential line and that of the posterior corneal surface with its apex in the angle recess; the angle-opening distance at 500 µm (AOD500) and AOD at 750 µm (AOD750), defined as the distance of a perpendicular line from the trabecular meshwork on the iris at point 500 or 750 µm from the sclera spur; and the trabecular-iris space area up to 500 µm (TISA500) (Figure 1A) or 750 µm (TISA750) (Figure 1B), defined the area bounded by the corneal endothelium, trabecular meshwork, and anterior iris surface out to a distance of 500 or 750 µm from the scleral spur (Figure 1).

Statistical analysis was performed using IBM SPSS Statistics for Windows (version 22.0; Armonk, NY). Descriptive statistics are presented as mean ± SD. A ꭓ2 test was used to evaluate the differences in categorical data. Continuous data were analyzed by the Wilcoxon test or logistic regression analysis when appropriate. All p values relate to two-sided tests with a significance level of p = 0.05.

## Results

A total of 42 eyes from 42 patients (19 males and 23 females), with an average age of 71.85 ± 5.3 years, were enrolled in this study. All recruited patients were Caucasians. Out of the study population, 22 patients were analyzed as the control group along with 20 patients with POAG. Baseline characteristics are summarized in Table 1.

**Table 1:** Baseline characteristics of the study population. POAG, primary open-angle glaucoma; CCT, central corneal thickness; AL, axial length The data are presented as 'mean±SD' and 'n'.

	Control group	POAG group	p value
Patients	22	20	
Age (years)	72.31 ± 6.25	70.18 ± 5.7	0.11
Pre-operative IOP	18.23 ± 3.76	13.82 ± 3.17	<0.01
Gender (male/female)	10/12	9/11	-
CCT (μm)	537.83 ± 33.11	524.26 ± 36.88	0.08
AL (mm)	23.96 ± 0.84	24.13 ± 0.62	0.3

**Figure 2:**
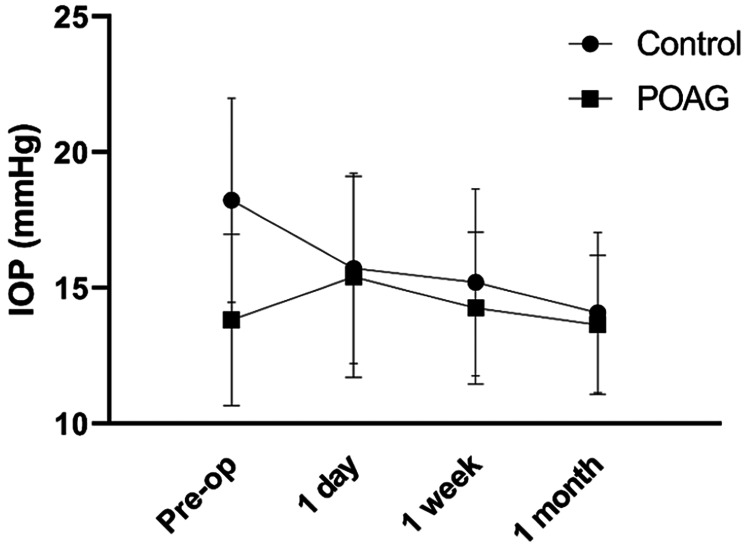
Line graph showing the IOP post-operatively and in follow-up visits of one day, one week, and one month after surgery in the control and the POAG group. IOP, intraocular pressure; POAG, primary open-angle glaucoma

**Figure 3:**
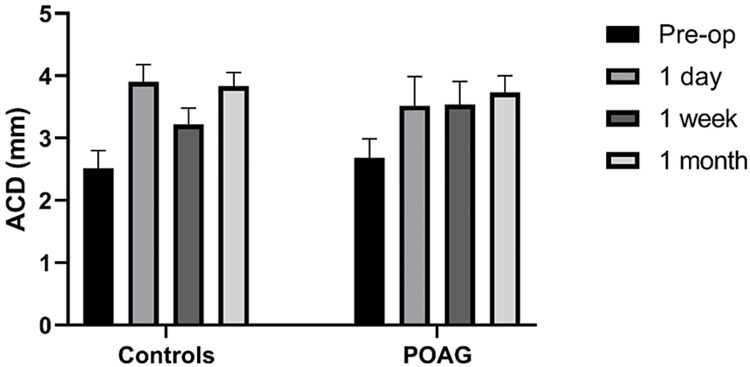
Box plots showing the ACD pre-operatively and in consecutive visits at one day, one week, and one month after surgery. The boxes are separated into the control and the POAG group. ACD, anterior chamber depth; Pre-op, pre-operatively; POAG, primary open-angle glaucoma

Consecutive measurements for IOP and ACD after one day, after one week, and after one month are shown in Figures 2-3. We observe that IOP (Figure 2) had a spike on day one in POAG patients (15.4 ± 3.71 mmHg) compared to pre-operative value (13.82 ± 3.17 mmHg), which reduced in value of 14.25 ± 2.81 mmHg in the follow-up visits as of week one and 13.64 ± 2.57 mmHg as of one month. In the control group, IOP reduction was immediate, one day following the operation (15.72 ± 3.58 mmHg) compared to pre-operative values (18.23 ± 3.76 mmHg), while it stabilized in even lower measurements of 15.21 ± 3.44 mmHg in week one and 14.08 ± 2.95 mmHg in month one.

ACD values showed significantly higher values for both groups, compared to pre-operative measurements (Figure 3). For the control group, ACD was 2.52 ± 0.28 mm pre-operatively, while it increased significantly one day following surgery (3.9 ± 0.33 mm), decreased to 3.22 ± 0.24 mm after one week, and reached its final value of 3.83 ± 0.28 mm one month after the operation. For the POAG group, ACD was measured at 2.68 ± 0.31 mm before surgery, while it increased to 3.52 ± 0.47 mm one day after the operation. It had similar values of 3.54 ± 0.37 mm in week one and stabilized at 3.73 ± 0.27 mm one month afterward.

For ACA, we observed a significant increase for both the control and the POAG group (p < 0.01) in pre-operative values compared to that one month afterward (Table 2). For AOD500 and AOD750, we can also see that there is a significant difference in pre- and post-surgery for both groups. For TISA500, there was not a significant difference for the control group (p = 0.056), while there was a significant increase in the POAG group (p < 0.01). Similarly, for TISA750, there was a statistically significant difference in the control group (p = 0.101), while we found a significant difference in the POAG group (p < 0.01).

**Table 2:** Anterior chamber and angle parameters before and after one month following cataract surgery in the eyes with POAG and normative data. *p values < 0.05; POAG, primary open-angle glaucoma; ACD, anterior chamber depth; ACA, anterior chamber angle; AOD, angle opening distance; TISA, trabecular iris space area The data are presented as 'mean±SD'.

	Control group			POAG group		
	Pre-operative	Post-operative	p value	Pre-operative	Post-operative	p value
IOP	18.23 ± 3.76	14 ± 3.58	0.01^*^	13.82 ± 3.17	13.64 ± 2.75	0.735
ACD	2.52 ± 0.23	3.83 ± 0.26	<0.01^*^	2.68 ± 0.31	3.73 ± 0.37	<0.01^*^
ACA	19.61 ± 5.31	29.65 ± 4.49	<0.01^*^	24.5 ± 4.35	33.58 ± 3.86	<0.01^*^
AOD_500_	300.72 ± 118.13	490.07 ± 140.12	<0.01^*^	410.77 ± 109.63	680.18 ± 160.47	<0.01^*^
AOD_750_	308.94 ± 101.53	719.79 ± 97.86	0.02^*^	429.21 ± 110.98	814.81 ± 107.62	<0.01^*^
TISA_500_	89.05 ± 32.12	115.51 ± 43.24	0.056	106.05 ± 30.06	188.45 ± 56.15	<0.01^*^
TISA_750_	170.12 ± 63.96	215.88 ± 85.71	0.101	192.76 ± 61.24	280.51 ± 90.84	<0.01^*^

## Discussion

The role of anterior segment parameters in the pathophysiology of glaucoma, including POAG, has gained increased attention. Post-phacoemulsification changes in these parameters, such as alterations in anterior chamber depth and angle width, might impact patients with existing glaucoma or those at risk of developing the condition. Modern imaging technologies such as AS-OCT have revolutionized the detection and analysis of these changes. The capabilities of AS-OCT extend beyond these traditional areas, allowing for detailed examination of the anterior segment structures such as the iridocorneal angle, corneal thickness, intraocular lens position, and iris configuration [14,15].

In this study, we evaluated the short-term post-phacoemulsification changes of the anterior chamber using quantitative parameters measured by AS-OCT (RTVue XR Avanti). We found marked widening of the anterior chamber in both the control and the POAG group with significant ACD increase. Our findings are consistent with the observations made by Yang et al. [16], indicating a significant change in ACD following cataract surgery among subjects with normal eyes. Specifically, individuals who underwent cataract surgery exhibited a significant increase in ACD, angle opening distance at 500 mm, and anterior chamber area as well as a significant reduction in IOP. Poley et al. [17] also reported that cataract surgery significantly increased the ACD in eyes with angle-closure glaucoma, open-angle glaucoma, and control eyes (normal IOP).

A study by Day et al. [18] found that ACD is a significant risk factor for POAG. This relationship is thought to be due to the anatomical configuration that predisposes individuals to increased IOP, a major risk factor for POAG. Based on the presented work, we found that there is a significant IOP reduction in the control - naïve group; however, despite the lower measured IOP in the glaucoma group, there was no significant difference pre- and post-operatively.

Poley et al. [17] suggested that the magnitude of IOP reduction following phacoemulsification was positively related to the level of preoperative IOP, in both glaucomatous and non-glaucomatous eyes. Consistent with this finding, our study observed that the control group, which exhibited the highest preoperative IOPs, experienced the greatest reduction in IOP. Moreover, recent research has indicated that the post-operative reduction in IOP in eyes with wide anterior chamber angles could be attributed to several mechanisms. Phacoemulsification has been demonstrated to enhance trabecular outflow facility, resulting in decreased IOP [19]. However, the precise mechanism underlying this enhancement in the outflow facility remains unclear. More recently, Johnstone et al. [20] proposed that cataract surgery, by deepening the anterior chamber and inducing scleral expansion, influences ciliary muscle tension and IOP through alterations in the position and tension of the ciliary muscle, trabecular meshwork (TM), and scleral spur attachments. This process ultimately leads to the enlargement of TM interspaces and an increase in the lumen volume of Schlemm's canal, resulting in improved outflow dynamics. Interestingly, there is a spike on the first post-operative day in the POAG group (Figure 2), which stabilized in the follow-up examinations. The mechanism of the increase in the acute post-operative period is unclear. Risk factors include remnants of ocular viscoelastic agents, intraocular inflammation, or mechanical deformation of angle structures [21-23].

Moreover, in this study, we found that ACD increases rapidly on day one after phacoemulsification, while in the POAG group, the anterior chamber tends to widen slower (Figure 3).

We also showed that angle volume as measured by the sensitive AOD and TISA indices [24,25] can have significant changes following cataract surgery. While AOD was significantly increased in both the control and the POAG group, TISA500 and TISA750, while having increased values for both groups, only reached statistical significance in the POAG group. This could depend on the specific iris and angle configuration and their alterations following cataract extraction. The exact reason remains unclear, and there are confounding parameters to be considered such as iris pigmentation and pupil size [26-28].

A possible limitation of this study is that this is a single-center study with a lack of ethnic diversity. Our study involved a relatively small sample size of glaucoma patients undergoing phacoemulsification, which could increase the risk of type 2 errors. Patient follow-up was also limited to one month post-operative. Therefore, the study might not be able to capture the long-term implications of cataract surgery on glaucoma progression or management. This study included patients who were already diagnosed with POAG; thus, it is not known whether cataract surgery could be protective for POAG development. Moreover, POAG patients were already under topical medication. IOP measurements and associated results are significantly affected by this fact.

## Conclusions

In conclusion, this study provides valuable insights into the impact of cataract surgery on anterior segment parameters in patients with glaucoma, specifically POAG, utilizing AS-OCT for detailed analysis, during the early post-operative period. Our findings demonstrate significant changes in anterior chamber depth and angle width post-phacoemulsification, suggesting potential benefits in glaucoma management. The increase in anterior chamber depth post-surgery, particularly noted in POAG patients, underscores the multifaceted relationship between anterior segment anatomy and glaucoma pathophysiology. Future research should aim to include a more diverse patient cohort and extend follow-up periods to fully understand the long-term implications of cataract surgery on glaucoma dynamics. This study reinforces the significant role of advanced imaging technologies in enhancing our understanding of glaucoma and tailoring interventions to optimize patient care.
